# Diphenhydramine Overdose: A Case Report and Topic Review of Prehospital Diagnosis and Treatment

**DOI:** 10.7759/cureus.70719

**Published:** 2024-10-02

**Authors:** Marshall A Frank, Jeremy Lund, David M Langley, Mitchell Annis, Tamas R Peredy

**Affiliations:** 1 Emergency Medicine, Florida State University College of Medicine, Sarasota, USA; 2 Toxicology, Sarasota Memorial Hospital, Sarasota, USA

**Keywords:** anticholinergic toxidrome, critical care transport, diphenhydramine overdose, lipid emulsion, out-of-hospital cardiac arrest, prehospital emergency medicine, seizure, sodium bicarbonate, wide complex tachycardia

## Abstract

Diphenhydramine (DPH), a readily available first-generation H1 receptor antihistamine, can have severe consequences when taken in excessive amounts and can lead to grave outcomes such as seizures, dysrhythmias, coma, and death. Recognizing the early signs and symptoms of DPH toxicity is crucial. Fortunately, fatal adult cases of DPH overdose are rare. This report describes a near-fatal overdose of a young adult female who experienced recurrent seizures, respiratory failure, and cardiac arrest with the return of spontaneous circulation (ROSC) in the prehospital setting and complete functional recovery. This case underscores the urgency of addressing DPH toxicity and the utility of reversal agents, such as sodium bicarbonate, in sodium channel blockade.

## Introduction

A vasoactive amine and inflammatory mediator, histamine has widespread physiological functions ranging from smooth muscle contraction (especially bronchial), vascular endothelial dilation, and atrioventricular nodal suppression to many peripheral nociceptive functions resulting in pain and pruritus [1]. Diphenhydramine (DPH) is an inverse agonist targeting histamine H1 receptors. It is available over the counter in different forms for allergies and colds and as a sleep aid. In 2015, antihistamines were ranked 14th on the list of medications associated with death, and among 92,033 overdose deaths during 2019-2020, 13,574 (14.7%) were antihistamine-positive, and 3,345 (3.6%) were antihistamine-involved; fewer than 0.1% (90) involved antihistamines alone [2,3].

Cardiac side effects range from mild tachycardia at therapeutic doses (due to its anticholinergic properties) to dysrhythmias. The dysrhythmias are primarily due to DPH directly affecting myocardial sodium (Na+) and potassium (K+) channels, leading to prolongation of the QRS and QT/QTc intervals observed on electrocardiography and are dose-dependent [1]. The neurological side effects of DPH are also dose-dependent and range from transient drowsiness and confusion to seizures, coma, or death, as it is lipophilic and readily crosses the blood-brain barrier. Although the exact dose of DPH associated with severe side effects is not known, many authors believe adults who ingest over 1 gram (g) have a heightened risk of seizures, coma, and death, and this is often referenced as the threshold of "toxicity" [4]. Overall, at toxic doses, neurological effects are more common than dysrhythmias. Seizures or the need for intubation are two predictors of severe outcomes, and the risk is even greater with ingestions over 1.5 g [5,6]. At therapeutic doses, the side effects of DPH are mild from its anticholinergic properties, as previously alluded to, and can include dilated pupils, mouth dryness, urinary retention, elevated body temperature, and dry skin [6].

Our primary concern in this case report is toxicity due to massive DPH ingestion; the key to stabilizing patients experiencing severe DPH toxicity by emergency medical service (EMS) personnel lies in a standardized approach to managing seizures and adhering to advanced cardiac life support (ACLS) guidelines [7], as large DPH ingestions are associated with ventricular tachycardia, seizures, and death [8,9]. A widened QRS complex with a large terminal R wave in lead AVR on electrocardiography due to sodium channel blockade is an early clue to recognizing substantial DPH cardiotoxicity. EMS and emergency department (ED) staff should consider early bicarbonate administration in the presence of this finding, which can prevent further clinical deterioration [7-9].

In this case report, a patient who thrice experienced cardiovascular collapse after massive DPH ingestion achieved full recovery following expeditious diagnosis and treatment. In light of this near-fatal outcome and remarkable recovery, we highlight the role of early bicarbonate therapy and advocate for its use in the prehospital management of sodium channel blockade. We also review lipid emulsion therapy as an emerging adjunctive treatment and other standard treatments.

## Case presentation

Emergency medical services brought a 24-year-old female to our ED after an intentional overdose. The patient was in her usual state of health until her family found her just before arrival unresponsive next to a half-full bottle of Sleep Aid™ (diphenhydramine HCl), which the patient had procured over the counter for the purpose suggested by the name. The ingestion was 182 × 25 milligram (mg) tablets (4.6 of DPH total, 69 mg/kilogram (kg)), per this patient's recorded weight of 66 kg.

Actions in the field

**Figure 1:**
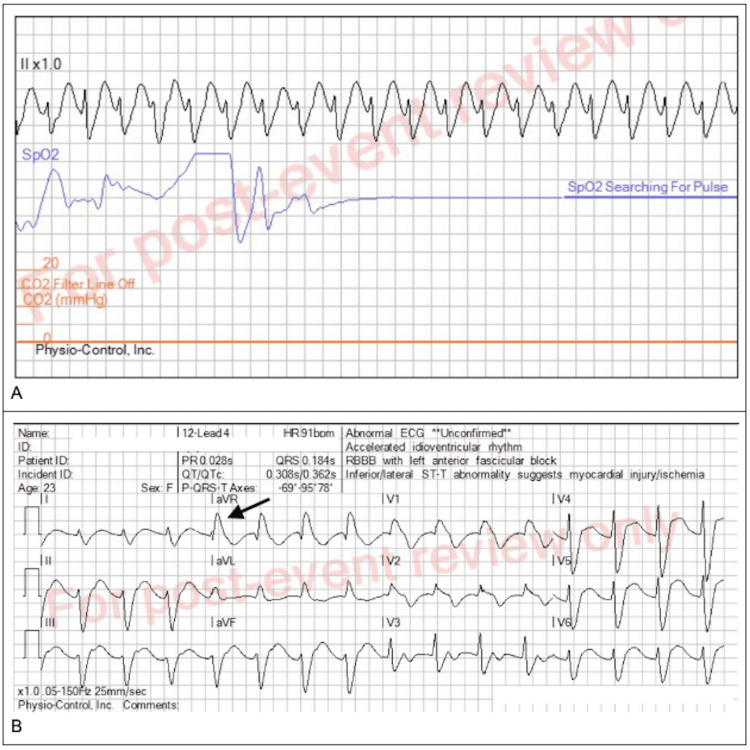
A: EMS rhythm strip obtained on arrival, demonstrating a wide complex tachycardia with a rate of 167 beats per minute. B: Prehospital EKG showing a wide QRS complex and large terminal R wave in lead aVR. Abbreviations: EMS: emergency medical service, EKG: electrocardiogram

EMS encountered the patient obtunded with purple vomitus and pill fragments over her face. The pulse was 167 beats per minute (bpm), there were 18 respirations per minute (rpm), and the end-tidal capnography was 29 millimeters mercury (mmHg) (range: 25-35 mmHg). Blood pressure was not recorded. The initial rhythm strip revealed a wide complex tachycardia (Figure 1A) at 144 bpm.

EMS started bag-valve-mask ventilation in anticipation of intubation. She experienced three generalized seizures for which 2.5 mg of lorazepam was given intravenously (IV). Postictally, the blood pressure was 135/105 mmHg, oxygen saturation was 87% on room air, and respiratory rate was 18 rpm. An electrocardiogram (EKG) showed a wide complex rhythm (rate of 91 bpm) with a prominent terminal R wave in lead aVR (Figure 1B).

She was intubated after rapid sequence induction (RSI) via direct laryngoscopy with a 6.0 endotracheal tube (ETT) using 150 mg of ketamine IV and 120 mg succinylcholine IV, with subsequent nasogastric tube placement. During transport, she decompensated to the point of cardiac arrest with pulseless electrical activity. Before ED arrival, the patient underwent 16 minutes of cardiopulmonary resuscitation (CPR) and was given three 1 mg doses of 1:10,000 epinephrine IV and 1 g of calcium chloride IV, leading to a return of spontaneous circulation (ROSC). She required two (2) additional aliquots of push-dose epinephrine 1:100,000 IV en route for hypotension.

Actions in the emergency department

The patient arrived at the ED intubated and sedated after 20 minutes of transit. Her initial axillary temperature was 97.8°F, heart rate (HR) was 87 bpm, blood pressure was 57/26 mmHg, and fingerstick glucose was 134 milligrams/deciliter (mg/dL). She had an oxygen saturation of 99% on continuous mandatory ventilation (CMV) with a set respiratory rate of 23 rpm, a tidal volume of 300 milliliter (mL), and a positive end-expiratory pressure of 8 cm H2O with a fraction of inspired oxygen (FIO2) of 100%. Her physical examination was notable for vomitus over the face and neck, minimally reactive pupils (5 mm), dry skin, and no response to noxious stimuli.

**Figure 2:**
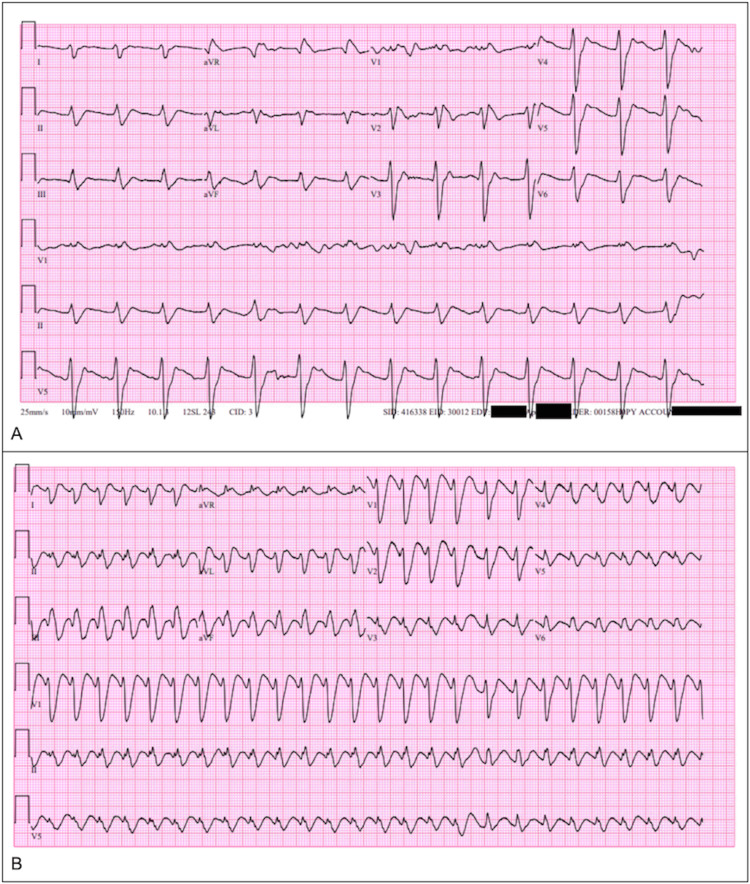
A: Initial EKG with a wide complex rhythm, QRS 174 ms, QTc 611, and HR of 87 bpm. B: EKG prior to second shock delivery with an HR of 155 bpm, QRS 162 ms, and QTc 491 ms. Abbreviations: EKG: electrocardiogram, HR: heart rate

Her initial EKG (Figure 2A) demonstrated a wide complex rhythm with a QRS of 174 milliseconds (ms) and QTc of 611 ms at 87 bpm, suggesting sodium channel blockade from DPH toxicity. She received a bolus of 150 milliequivalents (mEq) of sodium bicarbonate and a standard concentration infusion of sodium bicarbonate (150 mEq sodium bicarbonate diluted in 850 mL 5% dextrose in water) started at 200 milliliter/hour (mL/hour). In addition, she received a liter of lactated Ringer's crystalloid solution.

Thirty minutes after arrival in the ED, the cardiac monitor displayed ventricular tachycardia. Immediate CPR and defibrillation resulted in ROSC within minutes.

Ten minutes later, after another rhythm change, an EKG demonstrated an HR of 155 bpm, QRS duration of 162 ms, and QTc of 491 ms (Figure 2B), suspicious for recurrent ventricular tachycardia.

Another round of CPR and defibrillation resulted in ROSC. Her initial relevant laboratory studies resulted shortly after revealing a mild anion gap acidosis and hypokalemia, with other pertinent studies in Table 1 for reference.

**Table 1:** Initial laboratory values. Abbreviations: ng/L: nanograms/liter, mmol/L: millimoles/liter, mg/dL: milligrams/deciliter, gm/dL: grams/deciliter, uIU/L: micro-international units per milliliter, U/L: units per liter, BUN: blood urea nitrogen, AST: aspartate transaminase, ALT: alanine transaminase, INR: international normalized ratio

Test	Result	Reference range	Units
Glucose	234	70-100	mg/dL
Sodium	136	132-144	mmol/L
Potassium	2.6	3.5-5.1	mmol/L
Chloride	109	98-110	mmol/L
Carbon dioxide	15	21-32	mmol/L
BUN	9	6-20	mg/dL
Creatinine	1.21	0.70-1.30	mg/dL
Calcium	11.4	8.3-10.2	mg/dL
BUN/creatinine ratio	7	8-20	N/A
Magnesium	2.7	1.6-2.5	mg/dL
Ethanol	<0.010	0.000-0.010	gm/dL
Thyroid-stimulating hormone	2.130	0.358-3.740	ulU/mL
Acetaminophen	<2	10-30	ug/mL
Salicylate	<1.7	2.8-20.0	mg/dL
Troponin	5	0-59	ng/L
Creatine kinase	95	39-308	U/L
AST	16	15-37	U/L
ALT	16	13-56	U/L
Alkaline phosphatase	52	33-149	U/L
Direct bilirubin	0.1	0.0-0.3	mg/dL
Total bilirubin	1.1	0.2-1.3	mg/dL
Albumin	3.5	3.2-4.8	g/dL
Prothrombin time	11.8	9.2-11.7	Seconds
INR	1.16	0.88-1.13	N/A

She underwent an ETT exchange to clear pill fragments clogging the tube. Gastric suctioning was performed with a 16 French orogastric tube (OGT), removing copious sediment. Toxicology advised activated charcoal delivery and post-arrest lipid emulsion therapy. A lipid emulsion bolus of 20% of 95 mL (1.5 mL/kg) was administered with an infusion initiated at 0.025 mL/kg/minute for one hour. The arterial blood gas results drawn post-cardiac arrest ETT exchange and sodium bicarbonate infusion are in Table 2, revealing a decreased pH, significant lactic acidosis, and mild hypercarbia.

**Table 2:** Post-arrest arterial blood gas. Abbreviations: mmol/L: millimoles/liter, mEq/L: milliequivalents/liter, millimeters mercury: mmHg, PaCO2: partial pressure of carbon dioxide, PaO2: partial pressure of oxygen, HCO3: bicarbonate

Test	Result	Reference range	Units
pH	7.20	7.35-7.45	N/A
PaCO2	50	35-45	mmHg
PaO2	402	80-100	mmHg
HCO3	30	22-26	mEq/L
Lactic acid	7.8	0.9-1.9	mmol/L

The pulmonary critical care team performed a bronchoscopy in the ED, successfully removing additional pill fragments predominantly from the right upper, middle, and lower lobes. Resuscitation continued with lactated Ringer's solution at 200 mL/hour and sodium bicarbonate infusion at 150 mL/hour with potassium supplementation. The patient remained sedated with propofol at 12 microgram/kilogram/minute (mcg/kg/minute) and fentanyl at 25 mcg/hour. The patient was transported to the intensive care unit (ICU) on vasopressin and norepinephrine infusions at 0.04 units/minute and 38 mcg/minute, respectively. Vital signs just before transfer included a blood pressure of 158/89 mmHg, an HR of 146 bpm, a set respiratory rate of 30 rpm, and an oxygen saturation of 100% on 80% FIO2 via CMV.

Actions in the intensive care unit

**Figure 3:**
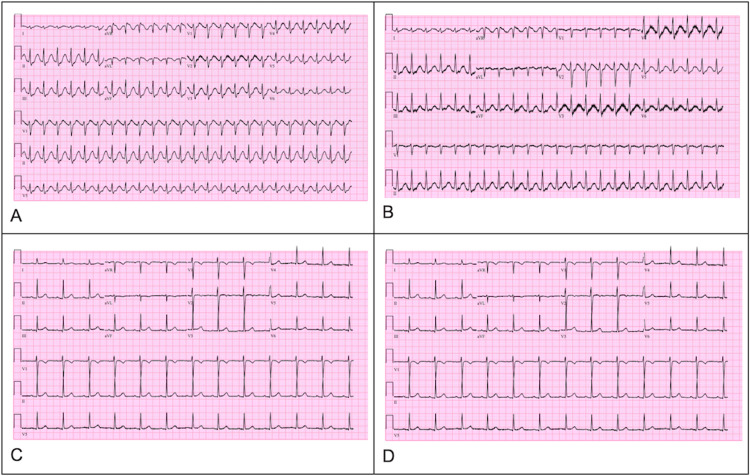
A: ICU day 1 EKG revealing narrowing of the QRS complex at 116 ms, QTc of 546 ms, and HR of 145 bpm. B: ICU day 2 EKG revealing further stability of the QRS complex at 118 ms, QTc of 588 ms, and HR of 137 bpm. C: ICU day 3 EKG revealing a QRS complex at 92 ms, QTc of 508 ms, and HR of 81 bpm. D: ICU day 4 EKG revealing a QRS complex at 92 ms, QTc of 429 ms, and HR of 76 bpm. Abbreviations: ICU: intensive care unit, EKG: electrocardiogram

The sodium bicarbonate infusion continued overnight. The following morning, a third EKG demonstrated a QRS complex of 116 ms, QTc of 546 ms, and HR of 145 bpm (Figure 3A). The patient's clinical status gradually improved with the stabilization of hemodynamics and narrowing of the QRS complex (Figure 3B). An EKG on the third day in the ICU revealed a QRS complex of 92 ms, QTc of 508 msec, and an HR of 81 bpm (Figure 3C). A repeat EKG before discharge demonstrated a QRS complex of 92 ms, QTc of 429 ms, and an HR of 76 bpm, indicating the resolution of the sodium channel blockade (Figure 3D). After five days of mechanical ventilation, the patient underwent extubation. She demonstrated excellent neurological recovery and was transferred to the behavioral health unit.

## Discussion

Prompt recognition of the constellation of symptoms associated with anticholinergic toxidrome is the key to prehospital care in suspected DPH ingestion. The mnemonic "mad as a hatter, dry as a bone, red as a beet, blind as a bat, hot as a hare, and full as a flask" may help recall the telltale clinical features. These symptoms, however, overlap with many other conditions, including sympathomimetic toxidrome (e.g., amphetamines and cocaine), central nervous system (CNS) depressant withdrawal (e.g., alcohol and benzodiazepines), postictal confusion, acute psychosis, and a myriad of medical conditions [10]. A complete set of vital signs and blood glucose is imperative to identify reversible causes of delirium. The absence of normal skin moisture and saliva are useful clinical clues. Stabilization of patients suffering from suspected DPH overdose by EMS personnel centers on two principles: management of neurological and cardiac manifestations of toxicity.

DPH inhibits central parasympathetic muscarinic pathways (autonomic and glandular) and disrupts average neurotransmitter balance. Benzodiazepines are considered first-line medications available to EMS personnel. These agents have potent anxiolytic, anticonvulsant, and sedative properties, all of which play a pivotal role in the management of DPH-induced agitation, delirium, and seizures. Lorazepam, for example, has a rapid onset and effective enhancement of gamma-aminobutyric acid-A (GABA-A) chloride channels and is the drug of choice for managing seizures and agitation [11]. Another rapid-onset choice is midazolam 5 mg, administered parenterally or intranasally. The standard dose of 5 mg IV may be repeated if needed. Benzodiazepines do not treat the underlying pathophysiology of the antimuscarinic effect. Physostigmine, a "true reversal agent," is favored in this toxidrome as it is a tertiary amine acetylcholinesterase inhibitor; however, it is unlikely to be readily available to EMS personnel. In the ED, this can be administered in 0.5-2 mg IV doses every 20 minutes, and if this is effective, 1-4 mg can be repeated at 30-60 minute intervals if seizures or deep coma recur. Its use in the setting of life-threatening dysrhythmias is controversial as there is some literature to suggest this may perpetuate further cardiac instability, so physostigmine was not used in our patient [11]. Other reversal therapies, such as intralipid emulsion therapy, have gained notoriety for lipophilic drug intoxications and can be given in a bolus dose of 1.5 mL/kg, followed by an infusion, but are not readily available to EMS staff [12].

Antipsychotic medications (e.g., haloperidol, droperidol, and olanzapine) used to control agitated delirium have anticholinergic properties and, in general, should be avoided. Ketamine is a dissociative anesthetic and may make behaviors more unpredictable.

In severe cases, hyperpyrexia (temperature > 105°F) may develop, an immediate life threat mandating emergent aggressive cooling. RSI-facilitated intubation with appropriate post-intubation analgosedation will reduce heat production and help facilitate further care and safe transport to the hospital.

Cardiotoxicity from DPH and other first-generation antihistamines can be seen on electrocardiography as QRS widening (>100 msec) due to sodium channel blockade, increasing the risk for ventricular tachycardia. Intravenous sodium bicarbonate is the first-line therapy to reverse the sodium channel blocking effects from various drug overdoses, including DPH [13,14]. The administration of sodium bicarbonate achieves a twofold effect. First, increasing the extracellular sodium concentration allows for a more favorable transmembrane gradient, and second, inducing a mild metabolic alkalosis can help reduce DPH's binding affinity to the sodium channels. These two effects work synergistically to narrow the QRS complex, allowing a more normal amount of sodium to pass through cardiac myocyte sodium channels and decreasing the likelihood of progression to ventricular arrhythmia.

In our case, the noteworthy findings on the prehospital 12-lead EKG are the large terminal R wave in lead aVR (Figure 1B) and widened QRS (>100 msec). There are several case reports of this finding associated with DPH toxicity [15]. QRS narrowing to reduce the risk of ventricular tachycardia involves boluses of 8.4% sodium bicarbonate 1-2 milliequivalents/kilogram (mEq/kg), rounded up to the next 50 mEq ampule size, with repeated doses as necessary based on clinical response and EKG monitoring [16,17]. Five minutes after each bolus dose, a repeat EKG should be obtained to assess the effectiveness of the dose in narrowing the QRS complex to 100 msec or less. A reasonable maximum of three bolus doses should be provided by EMS personnel to avoid excessive alkalemia or the inducement of hypokalemia [18]. In our case, a 24-year-old female presented with altered mental status and wide complex tachycardia that progressed to cardiogenic shock due to an overdose of DPH. The patient received approximately 400 mEq (6 mEq/kg) of 8.4% sodium bicarbonate in the ED and a sodium bicarbonate infusion at 150 mL/hour for approximately 16 hours.

In cases of cardiotoxicity progressing to stable ventricular arrhythmia refractory to sodium bicarbonate, the administration of IV lidocaine may serve as a rescue therapy. Lidocaine, a class 1b antiarrhythmic, selectively targets inactivated sodium channels, which are prevalent in ischemic and depolarized tissues. This selectivity allows lidocaine to reduce the arrhythmogenic potential of DPH without significantly prolonging the QRS complex or QT interval. Lidocaine possesses "quick on, quick off" kinetics, increasing the number of sodium channels that can conduct sodium [19]. Initial bolus doses of lidocaine typically range from 1 to 1.5 mg/kg [20]. Depending on the patient's response and the severity of the arrhythmia, a second dose may be administered.

One of this case's limitations is the lack of an initial blood pressure reading; if there was significant hypotension, this may have contributed to the peri-intubation arrest. It is also unknown to what degree the RSI agents used contributed to the patient's cardiac arrest in this particular overdose; although widely used agents, further study could be performed to determine the ideal agents for RSI in this scenario. Additionally, the lack of a tricyclic antidepressant (TCA) serum screening is perhaps an oversight due to the EKG similarities in TCA overdose to those seen in the setting of DPH overdose. Ultimately, however, multiple sources confirmed that the patient did not have access to this class of medications, including the patient who confirmed that she took Sleep Aid™ intentionally in this case and not a TCA. Furthermore, this is a single case report, and its generalizability is limited. The successful outcome for this patient cannot be applied broadly to other cases of DPH overdose, although it may be helpful as a point of reference for further studies.

## Conclusions

Our patient had a complete functional recovery. We believe that in light of the severe consequences, aggressive therapy, including upfront bicarbonate therapy, contributed to a good outcome. We advocate for consideration of early bicarbonate therapy for those who have overdosed on diphenhydramine with evidence of dysrhythmias, especially those with witnessed cardiac arrest by EMS. The routine use of sodium bicarbonate in prehospital care for patients in overdose situations with out-of-hospital cardiac arrest witnessed by EMS calls for further collaboration, research, and in-depth analysis of the topic.
